# Increased accuracy in diagnosing diverticulitis using predictive clinical factors

**DOI:** 10.48101/ujms.v127.8803

**Published:** 2022-12-15

**Authors:** Johanna Sigurdardottir, Abbas Chabok, Philippe Wagner, Maziar Nikberg

**Affiliations:** a Department of Colorectal Surgery, Västmanlands Hospital, Västerås, Sweden; b Centre for Clinical Research Region, Västmanland Uppsala University, Västerås, Sweden

**Keywords:** Colonic diverticulitis, C-reactive protein, diagnosis, diverticular disease, scoring system

## Abstract

**Background:**

The aim of this study was to identify clinical factors leading to increased diagnostic accuracy for acute colonic diverticulitis.

**Methods:**

Patients with clinical suspicion of acute colonic diverticulitis verified with computed tomography (CT) from two hospitals in Sweden between 9 January 2017 and 31 October 2017 were prospectively included. Symptoms, comorbidities, and laboratory results were documented. Candidate variables were analyzed using logistic regression, and the final variable set that yielded the most accurate predictions was identified using least absolute shrinkage and selection operator regression and evaluated using the area under the receiver operating characteristic (ROC) curve.

**Results:**

In total, 146 patients were included (73% women; median age 68 years; age range, 50–94 years). The clinical diagnostic accuracy was 70.5%. In the multiple logistic regression analysis, gender (female vs male odds ratio [OR]: 4.82; confidence interval [CI], 1.56–14.91), age (OR, 0.92; 95% CI, 0.87–0.98), pain on the lower left side of the abdomen (OR, 15.14; 95% CI, 2.65–86.58), and absence of vomiting (OR, 14.02; 95% CI, 2.90–67.88) were statistically significant and associated with the diagnosis of CT-verified diverticulitis. With seven predictors (age, gender, urinary symptoms, nausea, temperature, C-reactive protein, and pain left lower side), the area under the ROC curve was 0.82, and a formula was developed for calculating a risk score.

**Conclusion:**

We present a scoring system using common clinical variables that can be applied to patients with clinical suspicion of colonic diverticulitis to increase the diagnostic accuracy. The developed scoring system is available for free of charge at https://phille-wagner.shinyapps.io/Diverticulitis_risk_model/.

## Introduction

Diverticular disease is a common disorder in the Western world with over 50% prevalence in the 60 years and older age group (1). While it is among the most common causes for referrals to emergency departments (EDs) (2), only about 25% of patients have the classic triad of symptoms and laboratory results: abdominal pain, fever, and leukocytosis (3, 4). According to previous reports, the clinical diagnosis has an accuracy of 34–72% (5–9). The gold standard for diagnosing acute diverticulitis in the acute setting is laboratory work-up (white blood cell [WBC] count, neutrophilic count, and C-reactive protein [CRP]), and a computed tomography (CT) scan (10).

Several differential diagnoses, such as irritable bowel syndrome, inflammatory bowel disease, and colonic malignancy, are important to differentiate from acute diverticulitis. CT is currently the most commonly used radiological method for diagnosing acute diverticulitis with high sensitivity (95%) and specificity (96%) (11). CT reveals possible complications and can exclude other possible diagnoses (12). However, it exposes the patient to radiation, and the intravenous contrast used might be allergenic and nephrotoxic. With the incidence and prevalence of acute diverticulitis increasing in younger patients (13), the risk of numerous CT scans increases along with radiation exposure. As many of these patients are diagnosed and treated in an outpatient setting, it is important to differentiate acute diverticulitis from other diagnoses. Andeweg et al. (14) have proposed a scoring system for diagnosing acute colonic diverticulitis, and Lameris et al. (8) have developed decision rule for aiding a clinical diagnosis of acute diverticulitis. However, to our knowledge, no scoring system or decision rule is in frequent use (8, 14). The purpose of this study was to identify predictors that increase clinical accuracy in diagnosing acute diverticulitis and formulate a risk score (RS) to predict the probability of acute diverticulitis.

## Material and methods

A database was used from a previous study by Thorisson et al. (15), which focused on the radiological aspects of diagnosing acute colonic diverticulitis. The aim of that study was to evaluate whether non-enhanced low-dose CT was as sensitive as standard-dose CT in detecting acute colonic diverticulitis. That was a prospective observational study, conducted between 9 January 2017 and 31 October 2017 in two hospitals in Sweden (Västmanlands Hospital Västerås and Dalarnas Hospital Mora, a rural district hospital). Together, these hospitals have a catchment area of 340,000 people. Included patients underwent two CT scans (with and without contrast) and therefore included only patients 50 years and older. Patients with a clinical suspicion of acute diverticulitis were eligible for inclusion. The inclusion criteria were as follows: patients with clinical suspicion of acute diverticulitis, WBC count above 10 × 10⁹/L or CRP >25 mg/L (if WBC is not elevated), and age group 50 years and older. The exclusion criteria were as follows: pregnancy, contraindications to receive intravenous contrast medium such as renal failure or allergy, and patients who were unable for whatever reason to give informed consent (such as a language barrier or dementia). Patients who met the inclusion criteria were asked to participate. Inclusion was done in the ED as well as laboratory work-up and CT scans.

### Clinical evaluation of patients

On admission in the ED, symptoms were documented regarding abdominal pain; location of pain; anorexia; vomiting; changes in bowel movement; history of fever during the symptomatic period; duration of symptoms in hours or days; previous history of acute diverticulitis; comorbidities such as heart and vascular disease; lung, kidney or liver diseases; diabetes mellitus; or immunosuppression. The following results of the physical examination were documented by the on-call surgical resident on a data collection form: weight, height, vital parameters, signs of direct, indirect tenderness, and peritoneal irritation. The findings of the abdominal examination were documented on a four-grade intensity scale as none, slight, moderate, or strong tenderness. The patient was asked to mark his/her pain experience on a visual analogue scale (VAS) where 0 is none and 10 is the worst pain imaginable.

### Laboratory and radiological evaluation

WBC count, neutrophilic count, and the concentration of CRP were recorded.

After examination by a surgeon or a resident in surgery, all patients in the study underwent a CT scan with iodine-based intravenous contrast material with an individualized dosage based on the patient’s age, height, and weight. All CT scans were performed using a 64-slice General Electric Optima CT 1,600 machine (GE Healthcare, Marlborough, MA, USA) (15). The contrast medium used was Omnipaque™ (GE Healthcare) with a concentration of 350 mg iodine/ml (15). Findings on CT scans were assessed for signs of acute diverticulitis regarding inflamed diverticula of the colon, colonic wall thickness over 5 mm, pericolic fat stranding as well as complications due to acute diverticulitis and other diagnoses. Complications of acute diverticulitis were defined as abscesses (intramural, pericolic, or pelvic collections) or perforations (extra luminal gas located pericolic, retroperitoneal, or in the peritoneal cavity).

### Statistics

Means and standard deviations (SDs) were calculated for continuous variables. Descriptive odds ratios (ORs) generated by using standard logistic regression were used to quantify associations between independent variables and outcomes, both separate for each variable and combining all in a multivariable analysis. The area under the receiver operating characteristic (AUC-ROC) curve was calculated for each variable individually, including 95% confidence intervals (CIs). The risk factors were analyzed through univariable and multiple regression to evaluate the separate and combined contribution of these risk factors in predicting the risk of acute diverticulitis and in developing an RS.

L_1_ penalized logistic regression, least absolute shrinkage and selection operator (LASSO) was used to combine variables to create the RS, considering the relation between the number of events and number of variables, to prevent overfitting and reduce model complexity. All 14 variables were included in the initial analysis to select the variable set that yielded the most accurate predictions. A range of different penalties was used, and results were evaluated using 10-fold cross-validation of the AUC statistic and presented in a graph. The final variable set was also analyzed using an ordinary logistic regression model, and ORs, CIs, and *P*-values corresponding to a two-sided test of a null association with outcome were presented in Table 4. The sensitivity and specificity of different cut-offs for the RS resulting from the LASSO regression were also presented using a ROC curve and a corresponding standard AUC. The resulting formula for generating the RS for a patient is given together with a table for users to gauge the risk of diverticulitis for a patient, based on the score.

The analyses were done using statistical program R (16) and IBM SPSS Statistics (IBM Corp. Released 2016. IBM SPSS Statistics for Windows, Version 24.0. Armonk, NY, USA). *P*-values from two-tailed tests below the level of 0.05 were considered statistically significant.

## Results

### Study population

**Figure 1:**
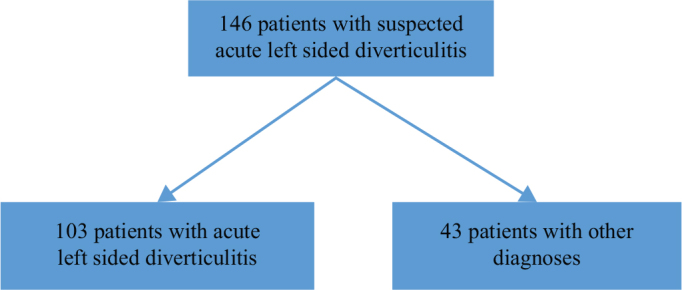
Flowchart of the cohort.

One hundred and forty-six patients were included in the study, all with clinical suspicion of acute diverticulitis. Figure 1 shows a flow-chart of the study population. The median age was 68 years (73% female: age range, 50–94 years). The mean body mass index was 28.6 kg/m^2^ (SD 4.6), and the mean symptom duration was 3.8 days (SD 3.7). The final diagnosis was based on clinical and radiological findings, as shown in Table 1. The three most common final diagnoses apart from acute diverticulitis were no cause found (12%), colitis (6%), and acute appendicitis (5%).

**Table 1:** Final diagnosis of patients suspected of having acute colonic diverticulitis (findings based on clinical and radiological results).

Final diagnosis	Number	%
Left-sided diverticulitis	103	70.5
Uncomplicated	82	
Complicated	21	
No cause found	17	11.6
Colitis	8	5.5
Appendicitis	7	4.8
Small bowel obstruction	3	2.1
Pyelonephritis/kidney stone	2	1.4
Basal pneumonia	1	0.7
Cholecystitis	1	0.7
Malignancy	1	0.7
Gynecologic cause	1	0.7
Spleen infarction	1	0.7
Perforation due to fishbone	1	0.7
Total	146	100

### Clinical evaluation for patients with acute diverticulitis

Diverticulitis was found in 103 of 146 patients with a diagnostic accuracy in terms of positive predictive value of 70.5%. Acute diverticulitis was complicated in 21 patients (20%), with contained perforations in 12, perforation with free air in three and abscess in six. The most common symptoms for patients with clinical suspicion for acute diverticulitis were left-sided abdominal pain (96%), nausea (33%), and change in bowel habits (obstipation 26% and loose bowels 25%), as shown in Table 2. A history of previous diverticulitis was found in 41 patients (40%). Most of the patients had a good Eastern Cooperative Oncology group performance status (0–1 in 66% of patients). The mean age in the acute diverticulitis group was 66 years (SD 9.1), compared with 70 years (SD 10.4) in the group with other diagnoses. History of pain duration for patients with acute diverticulitis was 3.6 days (SD 2.8) compared with 4.2 days (SD 5.2) for those with another diagnosis. A statistically significant difference between groups was seen in gender (female vs male OR, 4.82; 95% CI, 1.56–14.91), age (OR, 0.92; 95% CI, 0.87–0.98), pain vs no pain on the lower left side of the abdomen (OR, 15.14; 95% CI, 2.65–86.58), and absence vs presence of vomiting (OR, 14.02: 95% CI, 2.90–67.88). Table 3 shows the differences between groups.

**Table 2:** Patient characteristics and symptoms.

Variables	Total	Acute diverticulitis	Other diagnosis
	146	103 (70.5)	43 (29.5)
Gender			
Male	40	22	18
Female	106	81	25
Age group			8
50–59	40	32	8
60–69	41	30	11
70–79	50	34	16
80+	15	7	8
Previous acute diverticulitis*	53	41	12
Uncomplicated	45	35	10
Complicated	9	7	2
Comorbidity*			
Cardiovascular	46	33	13
Lung	12	8	4
Liver	2	2	0
DM	2	1	1
Kidney	1	0	1
Other	18	13	6
ECOG performance status			
0	119	86	33
1	14	10	4
2	8	4	4
3	2	1	1
4	0	0	0
Missing	3	2	1
Symptoms*			
Pain left side	141	101	40
Pain right side	5	2	3
Obstipation	38	27	11
Diarrhea	36	24	12
Nausea	48	32	16
Vomiting	16	4	12
Urinary symptoms	12	10	2
VAS scale			
0–4	29	19	10
5–7	65	51	14
8–10	32	21	11
Missing	20	12	8
BMI (kg/m^2^, *n* = 131)^#^	28.6 (4.6)	29.0 (4.6)	27.8 (4.3)
Clinical signs			
Body temperature, °C^#^	37.4 (0.7)	37.5 (0.7)	37.1 (0.7)
Localization of abdominal tenderness*			
Left lower quadrant	129	93	36
Suprapubic	68	53	15
Left right quadrant	40	25	15
Rebound tenderness			
None	37	22	15
Minimal	62	44	18
Medium	36	29	7
Severe	11	8	3
Laboratory results			
WBC (×10⁹/L)^#^	11.8 (4.4)	11.6 (3.2)	12.3 (6.5)
Neutrophiles (×10^9^/L) (*n* = 134)^#^	8.7 (3.2)	8.67(3.0)	8.9 (3.7)
CRP (mg/L)^#^	82 (61)	90 (60)	63 (62)

* Patients can have a history of both uncomplicated and complicated AD, more than one comorbidity, more than one symptom, and pain can be in more than one location on the physical examination.

# Continuous values are presented as mean (standard deviation).

Other values in number of patients and parentheses are percentages.

ECOG: Eastern Cooperative Oncology Group; VAS: Visual Analogue Scale; BMI: Body mass index; WBC: White blood cell count; CRP: C-reactive protein.

**Table 3:** Symptoms and physical examination. The diagnostic value of acute diverticulitis using odds ratios (ORs) with 95% confidence intervals (CIs) and logistic regression.

Patient characteristics		Simple regression analysis		Multiple regression analysis	
	Total *N* = 146	OR (95% CI)	AUC (%) (95% CI)	OR (95% CI)	*P*
Gender					
Female	106	2.65 (1.23–5.71)	0.60 (0.50–0.71)	4.82 (1.56–1,491)	0.01
Male	40	1.00 (reference)		1.00 (reference)	
BMI (*n* = 90)^#^	29 (4.6)	1.06 (0.97–1.16)	0.57 (0.46–0.67)		
Age^#^	68 (9.7)	0.95 (0.92–0.99)	0.63 (0.53–0.73)	0.92 (0.87–0.98)	0.01
Previous diverticulitis					
No	93	1.00 (reference)	0.56 (0.46–0.66)		
Yes	53	1.71 (0.79–3.71)			
Symptoms					
Pain lower left side					
No	9	1.00 (reference)	0.56 (0.45–0.66)	1.00 (reference)	<0.001
Yes	137	5.41 (1.29–22.73)		15.14 (2.65–86.58)	
Nausea					
No	110	1.00 (reference)	0.56 (0.46–0.66)	1.00 (reference)	0.58
Yes	36	2.02 (0.81–5.04)		1.44 (0.39–5.28)	
Obstipation					
No	108	1.00 (reference)	0.50 (0.40–0.61)	1.00 (reference)	0.09
Yes	38	1.03 (0.46–2.33)		3.05 (0.83–11.25)	
Diarrhea					
No	110	1.00 (reference)	0.52 (0.42–0.63)	1.00 (reference)	0.44
Yes	36	0.79 (0.35–1.76)		0.64 (0.21–1.98)	
Urinary symptoms					
No	134	1.00 (reference)	0.53 (0.42–0.63)	1.00 (reference)	0.22
Yes	12	2.20 (0.46–10.50)		4.28 (0.43–42.63)	
Vomiting					
No	130	9.58 (2.88–31.85)	0.62 (0.51–0.73)	14.02 (2.90–67.88)	<0.001
Yes	16	1.00 (reference)		1.00 (reference)	
VAS (*n* = 91)^#^	6 (2.2)	1.01 (0.84–1.20)	0.49 (0.37–0.61)		
Clinical signs					
Body temperature, °C^#^	37.4 (0.7)	2.07 (1.21–3.52)	0.65 (0.55–0.75)		
Rebound tenderness VAS					
None	37	1.00 (reference)	0.60 (0.50–0.70)	1.00 (reference)	0.84
Minimal	62	1.67 (0.71–3.92)			
Medium	36	2.82 (0.98–8.11)		1.06 (0.60–1.88)	
Severe	11	1.81 (0.41–7.99)			
Laboratory results^#^					
WBC ×10^9^/L	11.4 (4.4)	0.97 (0.89–1.04)	0.50 (0.39–0.61)	0.91 (0.82–1.01)	0.09
Neutrophiles ×10^9^/L	8.6 (3.2)	0.97 (0.86–1.09)	0.49 (0.36–0.61)		
CRP (>5) mg/L	71 (61)	1.01 (1.00–1.02)	0.65 (0.56–0.76)		

# Continuous values are presented as mean (standard deviation) in the total column.

Other values are presented in number of patients.

BMI: Body mass index; VAS: Visual Analogue Scale; WBC: White blood cell count; CRP: C-reactive protein.

**Table 4:** Table for translating risk scores into risk of acute diverticulitis in a clinical population of patients with suspected diverticulitis.

Risk score	Risk (%)
–2.94	5
–2.20	10
–1.73	15
–1.39	20
–1.10	25
–0.85	30
–0.62	35
–0.41	40
–0.20	45
0	50
0.20	55
0.41	60
0.62	65
0.85	70
1.10	75
1.39	80
1.73	85
2.20	90
2.94	95

### Predictor selection and evaluation

**Figure 2:**
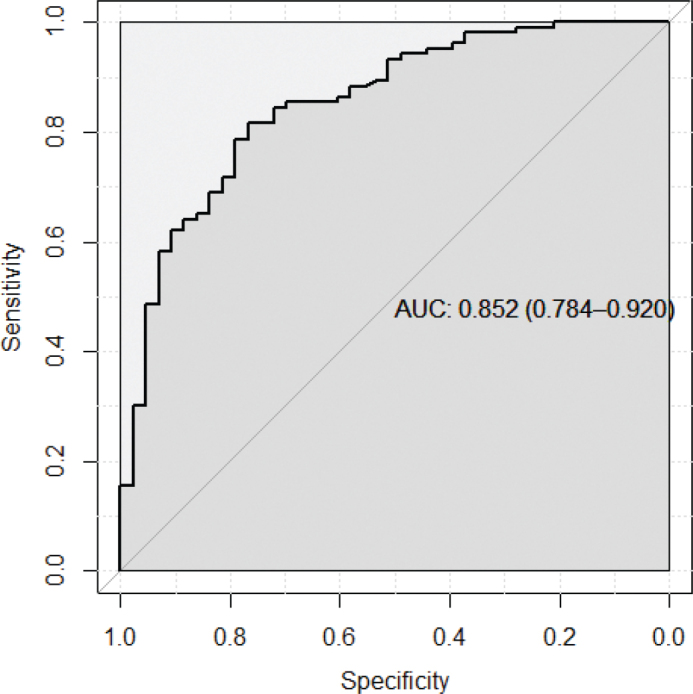
Area under the receiver operating characteristic curve for the combined set of the seven best predictors of acute diverticulitis (age, gender, urinary symptoms, nausea, temperature in degrees Celsius, CRP >5, and pain on the lower left side).

**Figure 3:**
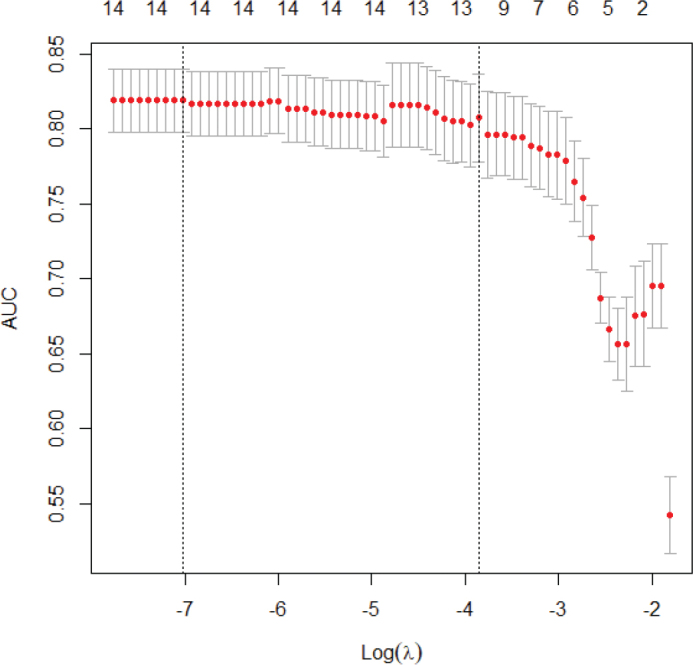
Least absolute shrinkage and selection operator (LASSO) regression with up to 14 variables. The area under the receiver operating characteristic curve decreases as the logarithm of the penalty λ increases and the variables become fewer.

Variables with statistically significant AUC values were gender, absence of vomiting, moderate rebound tenderness, and CRP >5 (Table 3). The inclusion criteria for the laboratory results were WBC count > 10 × 10⁹/L and/or CRP >25 mg/L. To identify patients with only increased WBC count, we used the CRP value <5. To simplify presentation, and present risk increases, as opposed to risk decreases, we defined the variable as CRP >5 mg/L. CRP was also included in different sensitivity analyses with different cut-offs, as continuous and using regression splines, to determine which form yielded the most effective predictions. The discriminatory accuracy (DA) of each variable separately was low (AUC ranging between 0.60 and 0.65). Combining them using LASSO regression to select the best set of predictors yielded a considerable increase in DA with respect to diagnosing AD. The AUC observed was 0.82 using cross-validation and 0.88 (95% CI, 0.82–0.94) without using cross-validation, for 12 of 14 variables (age, gender, fever, nausea, obstipation, diarrhea, temperature, rebound tenderness, CRP >5, WBC count, previous acute colonic diverticulitis, and pain on the left lower side of the abdomen). All combinations of the 14 variables using different penalties in the LASSO regression are shown in Figure 3. To get a more manageable and useful set of predictors, we settled for a set of seven predictors for the RS and a slight decrease in DA, to a cross-validated AUC of 0.82, corresponding to a standard AUC of 0.85 (0.78-0.92) as seen in Figure 2. The variables selected in the LASSO regression for this set were age, gender, urinary symptoms, nausea, temperature, CRP >5, and pain on the left lower side of the abdomen.

## Formula for generating the RS

The following formula was developed for calculating the RS for acute diverticulitis:


$$\begin{array}{c} \mathrm{RS}=-14.6+0.65*\mathrm{gender}-0.029*\mathrm{age}+0.18*\mathrm{urinary}+1.48\\ {}*\;\mathrm{vomit}+0.34*\mathrm{temp}+1.86*\mathrm{CRP}5+1.36*\mathrm{tender} \end{array}$$

where *gender* takes the value of 1 for women and 0 for men, *age* is the patient age in years, *urinary* takes the value of 1 if the patient has urinary symptoms and 0 if they do not, *vomit* takes the value of 1 if the patient has NOT vomited and 0 otherwise, *temp* is the patient’s temperature in degrees Celsius, *CRP5* takes on the value of 1 if the patient’s CRP is >5, and *tender* takes on the value of 1 if there is a pain on the left lower side. In Table 4, the RS gives the likelihood of acute diverticulitis based on the variables.

## Discussion

The current study proposes that usage of common symptoms such as left-sided abdominal pain, gender, age, change in bowel habits, and absence of vomiting may improve diagnosis in patients with clinical suspicion of acute diverticulitis. The high diagnostic accuracy in terms of the AUC at 0.82 found in this study indicates that even in a selected population of patients with clinical suspicion of diverticulitis, factors remain that facilitate improved diagnostic accuracy.

Andeweg et al. (14) proposed a scoring system with several predictive clinical and laboratory factors. We used their nomogram on our cohort and found an AUC of 0.70, i.e. a fair ability to differentiate between those with verified acute diverticulitis and those identified as having other diagnoses. By using our scoring system, the accuracy of the diagnosis for our cohort was increased further to reach 0.79.

However, Andeweg’s nomogram was developed for use in a different clinical setting and patient cohort, a major difference being that our cohort consists of patients with clinical suspicion of left-sided acute diverticulitis. Therefore, we hypothesized and subsequently verified that accuracy could be increased if we created an RS tailored to this setting.

In Sweden, patients with abdominal pain that require work-up are either referred to the ED by their general practitioner or attending ED directly. All patients are evaluated by an on-call physician (often surgeon or surgical resident), and most undergo a diagnostic CT scan with contrast as well as laboratory work-up; the present study is consistent with this approach.

A strength in this study is that each patient had a CT scan, which was re-evaluated by an experienced gastrointestinal radiologist, verifying that the included patients were correctly diagnosed. However, some patients with acute diverticulitis may not have been included in the study. There are limitations with this study. One of which is a limited and selected cohort and the need for the scoring system to be validated before being used. We have tried to address these concerns in the text below. We used the LASSO regression to select relevant variables for our RS and to estimate their association with outcome (17, 18). This was done because of our somewhat limited sample size, proportion of non-events, and the number of potential variables to consider when generating the RS. Since we attempted this analysis using a regular regression approach, we would likely have to run the risk of overfitting our RS to our study population and limited the ability to generalize these findings and the use of the score in other patient settings. An indication of overfitting was the difference between the cross-validated and the regular AUC, where the former is more robust to these issues. However, we cannot rule out, even with our more robust approach, that results were affected to some extent by overfitting bias, even if it should have mitigated most of these issues.

Another limitation of the study is the selected cohort. When interpreting results from the present study, it is important to keep in mind that the study population is one where acute diverticulitis is already suspected, as is seen in the clinical diagnostic accuracy of 70%, which is higher than that in other studies (8, 19). Because of this bias, variables selected for predicting a verified acute diverticulitis diagnosis may not be the same as in other studies conducted in a more general clinical population or without a suspected clinical acute diverticulitis diagnosis. The difference here may be that variables on which the clinical suspicion of acute diverticulitis is based may have reduced variability in our cohort compared with more general clinical populations, which makes these variables less useful for prediction in our cohort. One such example may be pain in the left lower quadrant, which is likely to be present in most clinically suspected cases of acute diverticulitis. The fact that it is one of the symptoms also indicates that it is a useful predictor of acute diverticulitis in a general clinical population, as those with pain in the left lower quadrant are much more likely to have a verified acute diverticulitis than are those who do not. The fact that lower left quadrant pain was prevalent in our cohort, as well as associated with a large difference in risk, is evident from Table 3, where we can see that pain in the left lower side substantially increases the probability of the patient having acute diverticulitis. However, as this variable is present in most cases, it also is a poor predictor of acute diverticulitis in our cohort, as is clear from the rather low AUC value.

A somewhat surprising result of the present study was that the limit of CRP to produce the best prediction of verified acute diverticulitis was as low as 5. One would possibly expect the risk of acute diverticulitis to increase with increasing CRP. However, in the present study, where many with suspected acute diverticulitis have elevated CRP, average CRP levels may not substantially differ between those with and without a confirmed acute diverticulitis diagnosis. Instead, a more useful difference may be found in those patients with CRP <5, where all but one did not have acute diverticulitis. This is similar to the reasoning above, where studying only suspected cases changes the distribution and association of known predictors to outcome.

Acute diverticulitis is a common diagnosis, and the gold standard for confirming the diagnosis in these patients is a CT scan. The scoring system, when validated in other cohorts, is not intended to be used instead of a radiological diagnosis, but rather, to be used as a complementary tool in the diagnostic arsenal. Not all centers have access to a CT scan all hours of the day and may need to refer their patients to get a CT scan. In some cases, the patients need to travel to do a CT scan. In those cases, if the clinician has a high suspicion of AD and the patient is clinically stable, this scoring system may aid in the decision to do a CT scan and when. However, we impress upon the fact that a scoring system does not differentiate between diagnoses as a CT scan does, and the patient’s clinical status is the dominant factor in deciding when and how the diagnosis is done. The authors emphasize that the scoring system is not a guarantee for the diagnosis ‘acute diverticulitis’ but a calculation of the risk of having acute diverticulitis. This is important to remember when using the system. Another instance where this system may be in aid of diagnosing diverticulitis is for patients with recurring acute diverticulitis presenting numerous times with similar symptoms. If clinically stable, perhaps these patients need not do a CT scan each time, and with aid from the scoring system, a more selective approach can be undertaken. This was a selected group of patients with clinical suspicion of acute diverticulitis. We sought to design a scoring system for AD to increase the clinical diagnostic accuracy. The developed scoring system is available for free at https://phille-wagner.shinyapps.io/Diverticulitis_risk_model/.

## Conclusion

Common symptoms and clinical findings can predict and improve diagnosis in patients with suspicion of acute diverticulitis. We present a scoring system that can increase the diagnostic accuracy of acute diverticulitis and may lead to a reduction of repeated CT scans.

## Ethics approval

This study was approved by the regional ethics committee and followed the 2013 Declaration of Helsinki guidelines (registration number Dnr 2016/411 and Clinical trials registration number NTC03443011).

## Informed consent

Patients received oral and written information about the study and radiation risks. A written informed consent was required for participation.

## Conflict of interest

The authors declare they have no conflict of interest.
